# Prevalence, incidence and risk factors for anogenital warts in Sub Saharan Africa: a systematic review and meta analysis

**DOI:** 10.1186/1750-9378-8-27

**Published:** 2013-07-10

**Authors:** Cecily Banura, Florence M Mirembe, Jackson Orem, Anthony K Mbonye, Simon Kasasa, Edward K Mbidde

**Affiliations:** 1 Department of Child Health and Development Centre, Makerere University College of Health Sciences, Kampala, Uganda; 2 Department of Obstetrics and Gynecology, Makerere University College of Health Sciences, Kampala, Uganda; 3 Uganda Cancer Institute, P.O. Box 3935, Kampala, Uganda; 4 Department of Community Services, Ministry of Health, P.O. Box 7272, Kampala, Uganda; 5 School of Public Health, Makerere University College of Health Sciences, Kampala, Uganda; 6 Uganda Virus Research Institute, P.O. Box 49, Entebbe, Uganda

**Keywords:** Anogenital warts, Sub Saharan Africa, HIV, HPV vaccination

## Abstract

**Introduction:**

The quadrivalent HPV vaccine is highly effective in primary prevention of anogenital warts (AGWs). However, there is lack of systematic review in the literature of the epidemiology of AGWs in Sub Saharan Africa (SSA).

**Objective:**

To review the prevalence, incidence and risk factors for AGWs in SSA prior to the introduction of HPV vaccination programs.

**Methods:**

PubMed/MEDLINE, Africa Index Medicus and HINARI websites were searched for peer reviewed English language published medical literature on AGWs from January 1, 1984 to June 30, 2012. Relevant additional references cited in published papers were also evaluated for inclusion. For inclusion, the article had to meet the following criteria (1) original studies with estimated prevalence and/or incidence rates among men and/or women (2) detailed description of the study population (3) clinical or self-reported diagnosis of AGWs (4) HPV genotyping of histologically confirmed AGWs. The final analysis included 40 studies. Data across different studies were synthesized using descriptive statistics for various subgroups of females and males by geographical area. A meta - analysis of relative risk was conducted for studies that had data reported by HIV status.

**Results:**

The prevalence rates of clinical AGWs among sex workers and women with sexually transmitted diseases (STDs) or at high risk of sexually transmitted infection (STIs) range from 3.3% - 10.7% in East, 2.4% - 14.0% in Central and South, and 3.5% - 10.5% in West African regions. Among pregnant women, the prevalence rates range from 0.4% - 3.0% in East, 0.2% - 7.3% in Central and South and 2.9% in West African regions. Among men, the prevalence rates range from 3.5% - 4.5% in East, 4.8% - 6.0% in Central and South and 4.1% to 7.0% in West African regions. In all regions, the prevalence rates were significantly higher among HIV+ than HIV- women with an overall summary relative risk of 1.62 (95% CI: 143–1.82).

The incidence rates range from 1.1 – 2.7 per 100 person-years among women and 1.4 per 100 person years among men. Incidence rate was higher among HIV+ (3.0 per 100 person years) and uncircumcised men (1.7 per 100 person-years) than circumcised men (1.3 per 100 person-years).

HIV positivity was a risk factor for AGWs among both men and women. Other risk factors in women include presence of abnormal cervical cytology, co-infection with HPV 52, concurrent bacteria vaginoses and genital ulceration. Among men, other risk factors include cigarette smoking and lack of circumcision.

**Conclusions:**

AGWs are common among selected populations particularly HIV infected men and women. However, there is need for population-based studies that will guide policies on effective prevention, treatment and control of AGWs.

## Introduction

The epidemiology of AGWs in most of SSA is largely unknown since few studies have been conducted. Studies from high income countries show that the clinical burden has been increasing over the years since approximately 0.5-1.0% of adults below 50 years have AGWs [1-3].

Caused mainly by low-risk HPV type 6 and 11, AGWs affect both men and women [1]. They are highly infectious with about 65% of individuals with an infected partner developing lesions within 3 weeks [4]. The median time between infection and development of lesions is about 11–12 months among men [5,6] and about 5–6 months among women [7]. Rarely, AGWs have been associated with malignant Bushke-Lowenstein malignant tumors [8]. Their occurrence is strongly linked to HIV and weakly associated with cigarette smoking [1,4,5]. At present, the impact of highly active anti-retroviral therapy (HAART) remains unclear [5,6].

Except for Rwanda [9], most countries in SSA are yet to introduce or scale up HPV vaccination in national immunization programs. While reduction in disease burden due to HPV 16/18 may not be evident for decades, vaccination with the quadrivalent HPV vaccine should result in immediate measurable reduction in the incidence rate of AGWs. Preliminary results from the Australian national HPV vaccination program shows a significant decline in the number of cases of AGWs among the young vaccinated women and some herd-immunity effect in young unvaccinated heterosexual men [10]. This review was undertaken to assess the prevalence, incidence and risk factors for AGWs before the introduction of HPV vaccination programs in order to provide a basis for future program evaluation in SSA.

## Methods

### Identification and eligibility of relevant studies

PubMed/MEDLINE, Africa Index Medicus and HINARI websites were searched for peer reviewed English language published medical literature from January 1, 1984 up to June 30, 2012. The following Medical Subject Heading (MESH) and search terms were used alone or in combination “Sub Saharan Africa” AND (“anogenital warts” OR “venereal warts” OR “condylomata acuminata” OR “condylomata”) AND “risk factors”. Relevant additional references cited in published papers were also evaluated for inclusion. For inclusion, articles had to meet the following criteria (1) original studies with clear estimation of prevalence and/or incidence rates among men and/or women (2) detailed description of study population (3) clinical or self-reported diagnosis of AGWs (4) HPV genotyping of histologically confirmed AGWs. Studies focusing exclusively on case reports and commentaries were excluded.

### Data extraction

From each article, the following information was extracted: first author, publication journal name and year of publication, country of study population, study sample type (population- or clinic- based), study design, mean or median age with range/inter quartile range, whenever available, sample size, prevalence and/or incidence rate overall and by HIV status, whenever available, risk factors and the overall prevalence of HIV, whenever available. For studies that included populations from different countries, data was extracted separately for each country.

### Statistical analysis

Data across different studies were synthesized using descriptive statistics for different subgroups of females and males by geographical areas. A meta- analysis of relative risk was conducted for studies that had data reported by HIV status and results presented in a forest plot. In total, 40 studies (39 hospital - and 1 population-based) are included in this review.

## Results

The prevalence and incidence rates of AGWs in diverse female and male hospital-based study populations in East, Central and South, and West African regions is summarized in Tables 1, 2 and 3. Overall, there is inter- and intra-region variations in rates depending on the underlying prevalence rate of HIV-1 infection, study population and age group studied. While both young and adult populations were studied, there seems to be no trend or pattern of prevalence rates by age.

**Table 1:** Studies reporting prevalence of AGWs in women

**Author,**	**Country**	**Study design**	**Study population**	**Sample size**	**Mean or Median age**	**Prevalence of AGWs**^**2 **^**n (%)**	**Prevalence**	**Comments**
**Publication year**					**(years, range/IQR**^**1**^**)**		**of HIV-1 (%)**	
***East Africa***								
Kreiss et al., 1992 [11]	Kenya	^§^Cross-sectional	Sex workers	196	30.2 (HIV-1+) 31.5 (HIV-1-)	18/196 (9.2) Overall 15/145 (10.0) HIV-1+ 3/51 (6.0) HIV-1		
Fonck, et al., 2000 [12]	Kenya	"EntryTbl_st^§^Cross-sectional	Women attending STD^3^ clinic	520	26 ± 6.8 (14–49)	31/520 (6 .0) 5/520 (1.0)^a^	29.0	Prevalence of AGWs 5% (Non pregnant women) 9% (Pregnant women) 6% (One sexual partner)
Mayaud et al., 2001 [13]	Tanzania	^§^Cross-sectional	Pregnant women	660	23.4 ± 5.1 (15–44)	20/660 (3.0)	15.0	
Riedner et al., 2003 [14]	Tanzania	^§^Open cohort	Female bar workers	600	25.4	39/600 (6.5) Overall 39/408 (9.6) HIV + 0/192 (0.0) HIV -	68.0	
Namkinga et al., 2005 [15]	Tanzania	^§^Cross-sectional	Women presenting with complaints of genital infections	464		18/464 (3.9)	22.0	
Amone-P'Olak, 2005 [16]	Uganda	^‡^Cross-sectional	Formally abducted teenage girls in Northern Uganda	123	16.2 ± 2.2 (12–18)	67/123 (54.5)^a^		
Mbizvo et al., 2005 [17]	Tanzania	^§^Cross –sectional	Women seeking primary health care services	382	26.7 ± 6.0	8/382 (2.1)	11.5	
Msuya et al., 2006 [18]	Tanzania	^§^Cross-sectional	Women seeking reproductive health care services	382	24.6 (14–43)	7/382 (2.0)	6.9	
Riedner et al., 2006 [19]	Tanzania	^§^Serial cross-sectional	Female bar workers	600	25.5 (16–39)	5.2-10.7	67.0	
Aboud et al., 2008 [20]	TanzaniaMalawi and Zambia	^§^Cross-sectional	HIV-1 positive pregnant women	2292	(15–49)	195/2292 (8.5)		Prevalence of AGWs Blantyre – 42/474 (8.9) Lilongwe – 61/748 (8.2) Dar es Salaam – 31/428 (7.2) Lusaka – 61/642 (9.5)
Banura et al., 2008a [21]	Uganda	Baseline of a prospective cohort study	Young women attending a clinic for teenagers	1275	20 (12–24)	97/1275 (7.6)	8.6	
Banura et al., 2008b [22]	Uganda	^§^Baseline of a prospective cohort study	Pregnant women Attending ANC^5^	987	19 (14–24)	61/987 (6.2)	7.3	
Urassa et al., 2008 [23]	Tanzania	^§^Cross-sectional	Youth attending an STI^4^ clinic	214	20.2 (Females) (13–24) 21.5 (Males) (11–24)	7/214 (3.3)	15.3	HIV −1 prevalence in Males – 7.5%
Grijsen et al., 2008 [24]	Kenya	^§^Baseline of a prospective cohort study	Women at risk for HIV-infection	361	27 (23–32)	8/361 (2.4)	32.0	
Msuya et al., 2009 [18]	Tanzania	^§^Cross-sectional	Pregnant women	2655	24.6 (14–43)	11/2555 (0.4) Overall 2/184 (1.1) HIV + 9/2470 (0.4) HIV -	6.9	
Mapingure, et al., 2000 [25]	Tanzania	^§^Cross-sectional	Pregnant women	2654	24.6	34/2654 (1.3) 48/2654 (1.8)^b^	6.9	
***Central and South Africa***								
Latif et al., 1984 [26]	Zimbabwe	^§^Cross-sectional	Pregnant women attending STD clinic	175	22.3	23/175 (13.7)		
Mason et al., 1990 [27]	Zimbabwe	^§^Cross-sectional	Women attending STD clinic	100	(15–45)	14/100 (14.0) 1/59 (1.7)^a^		
Kristensen 1990 [28]	Malawi	^§^Cross sectional	Adult women with symptoms of STIs	16,218	26.8 ± 7.5	32/16,218 (0.2)	62.4	
Nzila et al., 1991 [29]	Democratic Republic of Congo	^§^Cross-sectional	Female sex workers	1233		30/1233 (2.4) Overall 21/431 (5.0) HIV + 8/802 (1.0) HIV-	35.0	
Le Bacq et al., 1993 [30]	Zimbabwe	^§^Cross-sectional	New STD clinic attendees	146		19/146 (13.0)	69.0	
Maher et al., 1995 [31]	Malawi	^§^Cross-sectional	Female patients in general medical care	61	31 (16–65)	6/61 (9.8)		
Taha et al., 1998 [32]	Malawi	^§^Serial cross-sectional surveys	Pregnant women	1990 – 6603 *HIV + 1502 HIV- 5101* 1993 – 2161 *HIV + 694 HIV- 1457* 1995 – 808 *HIV + 808 HIV- 701*		1990 1993 1995 Overall 4.8 3.1 2.5 HIV + 8.3 6.3 2.7 HIV- 2.2 1.7 1.0	23.0 (1990) 30.1 (1993) 32.6 (1995)	
Klaskala et al., 2005 [33]	Zambia	^§^Cross-sectional	Pregnant women	3160	25 ± 5.3 (14–43)	203/3160 (6.2)		
Mbizvo et al., 2005 [17]	Zimbabwe	^§^Cross –sectional	Women recruited from primary health care centers	386	26.5 ± 6.8	13/386 (3.4)	29.3	
Kurewa et al., 2010 [34]	Zimbabwe	^§^Cross-sectional	Pregnant women	691	24.2 ± 5.1	48/691 (7.0) 50 /691 (7.3)^a^	25.6	
Mapingure et al., 2010 [26]	Zimbabwe	^§^Cross-sectional	Pregnant women	691	24.2 ± 5.1	50/691 (7.3) 33/691 (4.8)^b^	25.6	
Menendez et al., 2010 [35]	Mozambique	^§^Cross- sectional	Women attending ANC and FP^6^ clinics and community	262	(14–61)	13/262 (5.0)	12.0	Prevalence of HIV-1 21.0% among FP clinic attendees
***West Africa***								
Oni et al., 1994 [36]	Nigeria	^§^Cross-sectional	STD clinic attendees	116		12/116 (10.5)		
Ghys et al., 1995 [37]	Ivory Cost	^§^Cross sectional	Female sexual workers	1209		105/1209 (8.7) Overall 79/567 (14.0) HIV + 26/642 (4.0) HIV -	80.0	
Meda et al., 1997 [38]	Burkina Faso	^§^Cross – sectional	Women attending ANC	645	25.3 ± 2.9 (15–41)	19/645 (2.9)		
Okesola et al., 2000 [39]	Nigeria	^§^Cross-sectional	Patients attending an STD clinic	861	(17–74)	68/861 (8.0)		
Bakare et al., 2002 [40]	Nigeria	^§^Cross-sectional	CSWs^7^ and women without symptoms of STIs			6.5 36.4^c^	34.3	
Domfeh et al., 2008 [41]	Ghana	^§^Cross-sectional	Women attending gynecological clinic	75	33.3 ± 9.2 (19–57)	4/75 (5.3)^a^		
Sagay et al., 2009 [42]	Nigeria	^§^Cross-sectional	Female sex workers	374	27.8 ± 6.7 (16–63)	17/374 (4.5)		Prevalence of AGWs 5/81 (6.1%) Lemon users 12/293 (4.1%) Non Lemon users
Jombo et al., 2009 [43]	Nigeria	^§^Cross- sectional	Patients with genital ulcer disease	699		369/699 (52.8) Overall 285/506 (56.4) HIV + 84/193 (43.6) HIV –		Prevalence *Males*: 13/329 (2.6%) *Females*: 8/177 (1.6%)
Low et al., 2011 [44]	Burkina Faso	^§^Baseline of Prospective cohort	CSWs and other women with high-risk sexual behaviors	765	28 (15–54)	27/765 (3.5) Overall 19/273 (7.0) HIV −1 + 8/492 (1.6) HIV -	34.9 HIV-1 0.7 HIV-1 &2	No prevalent AGWs among women on HAART

^a^ self-reported prevalence; ^b^ self-reported prevalence for the last 12 months; ^c^ self-reported prevalence among commercial sexual workers; ^1^Inter quartile range; ^2^Anogenital warts; ^3^Sexually transmitted disease; ^4^Sexually transmitted infection; ^5^Antenatal care; ^6^Family planning; ^7^Commercial sexual workers; ^§^ hospital-based study; ^‡^ Teenagers in an institution.

**Table 2:** Studies reporting AGWs in men

**Author, year**	**Country**	**Study design**	**Study population**	**Sample**	**Mean or Median age**	**Prevalence of AGWs**^**2 **^**(%)**	**Prevalence of HIV-1%**	**Comments**
				**size**	**(years, range/IQR**^**1**^**)**			
**East Africa**								
Grijsen et al., 2008 [24]	Kenya	^§^Baseline of a prospective cohort study	Men at high-risk for HIV infection	536	27 (24–33)	9/500 (1.8)	21.0	
Smith et al., 2010 [45]	Kenya	^§^Baseline of RCT^3^ on male circumcision	HIV negative sexually active men	2168	20 (19–28)	12/2168 (0.6) Overall 10/1089 (0.9) HIV + 2/1079 ( 0.2) HIV-		
Tobian et al., 2012 [46]	Uganda	^†^Cross-sectional	Heterosexual men	1399	15-49	23/1399 (1.6)^a^ Overall 16/421 (3.8)^a^ HIV + 7/978 (0.7)^a^ HIV –		
**Central and South Africa**								
Le Bacq et al., 1993 [31]	Zimbabwe	^§^Cross-sectional	New STD clinic attendees	319		39/319 (12.2)	61.0	
Maher et al. 1995 [32]	Malawi	^§^Cross-sectional	In-patient male patients in general medical care	62	39 (20–90)	3/62 (4.8)		
Machekano et al., 2000 [47]	Zimbabwe	^§^Baseline of prospective cohort study	Male factory workers who reported symptoms of STDs	374		22/374 (6.0)	20	
Müller et al., 2010 [48]	South Africa	^§^Cross-sectional	Heterosexual men attending sexual health services	214	29.8 ± 7.5	108/214 (50.5)	49.5	
**West Africa**								
Okesola et al., 2000 [40]	Nigeria	^§^Cross-sectional	STD^2^ clinic attendees	1,373	17-74	4.1		
Wade et al., 2005 [49]	Senegal	^§^Cross sectional	Men who have sex with men	463	18-52	13/463 (2.8)	18.1	21.5% Overall 0.5% HIV-2 2 2.9% HIV-1 & HIV-

^a^ Self-reported prevalence.

^1^Inter Quartile Range.

^2^Commercial sexual workers.

^3^Randomized Controlled Trial.

^§^hospital-based study.

^†^Population-based study.

**Table 3:** Studies reporting incidence rates of AGWs in men and women

**Author, year**	**Country**	**Study design**	**Study population**	**Sample**	**Mean or**	**Incidence rate/100**^**2**^	**HIV −1**	**Comments**
			**and site**	**size**	**median age**	**person-years of**	**prevalence%**	
					**(years, range)**	**AGWs**^**2**^		
**East Africa**								
Lavreys et al., 1999 [50]	Kenya	Prospective cohort	HIV negative truck drivers in Mombasa	746	26^a^ (17–58) 29^b^ (16–62)	1.4 overall 1.7 Uncircumcised 1.3 Circumcised		Annual incidence of HIV-1 – 3.0%
**West Africa**								
Ozumba et al., 1991 [51]	Nigeria	Retrospective cohort (1976–85)	Female STD^1^ clinic attendees	45	21 ( 5–36)	2.7 (range:1.6 – 3.6)		AGWs incidence highest among teenagers and students
Low et al., 2011 [44]	Burkina Faso	Prospective cohort	Female sex workers and other women at high risk	765	28 (15–54)	1.1 HIV -	34.9	HIV- 1 & HIV-2 prevalence 0.7%

^a^ uncircumcised men.

^b^ circumcised men.

^1^Sexually Transmitted Diseases.

^2^Anogenital warts.

### Prevalence rates among women by geographical region

The prevalence rates among women in East, Central and South, and West African regions are summarized in Tables 1. The prevalence rates of clinical AGWs among sex workers and women with STDs or at high risk for other STIs range from 3.3% - 10.7% in East, 2.4% - 14.0% in Central and South, and 3.5% - 10.5% in West African regions. Among pregnant women, the prevalence rates range from 0.4% - 3.0% in East, 0.2% - 7.3% in Central and South and 2.9% (single study) in West African regions.

### Prevalence rates of AGWs among men by geographical region

Eight (8) studies (3 East Africa, 2 Central and South Africa, 3 West Africa) reported prevalence rates of AGWs in men (Table 2). The prevalence rate among STD clinic attendees, men who have sex with men, and men with symptoms of STDs in Central and South and West African region range from 4.8% - 12.2% and 2.8% - 4.1%, respectively. The rates among men in the East African region range from 0.6 -1.8 percent.

### Prevalence rates by HIV status

**Figure 1:**
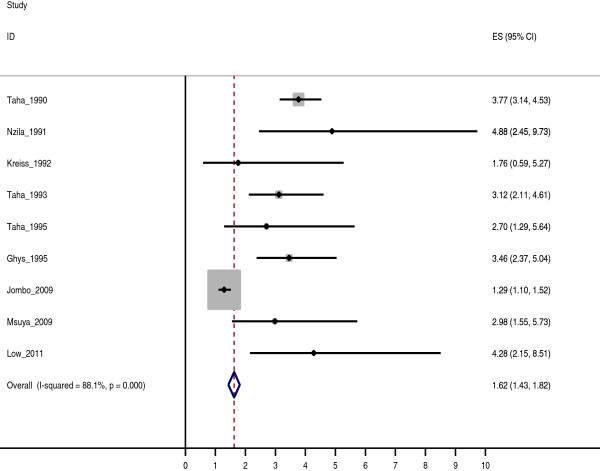
Relative Risk of AGWs among women with known HIV sero status.

The prevalence rates of AGWs were significantly higher among HIV+ than HIV- women in all regions with an overall summary relative risk of 1.62 (95% CI: 1.43–1.82) (Figure 1). Similarly among men, clinical and self -reported prevalence rates were higher among HIV+ than HIV- men (Table 2).

### Incidence rates of AGWs among men and women

Only 3 studies (2 among females and 1 among males) reported incidence rates of AGWs (Table 3). The incidence rates range from 1.1 – 2.7 per 100 person-years among women and 1.4 per 100 person years among men. The incidence rate was higher among uncircumcised (1.7 per 100 person-years) than circumcised men (1.3 per 100 person-years) [44].

### HPV 6 and/or 11 in AGWs

Only 3 studies reported the prevalence of HPV 6 and/or 11 in biopsy specimens or swabs taken from AGWs. HPV 11 was detected in 100% vulval-vaginal wart specimens obtained from 9 prepubescent South African girls [52]. HPV 6 and/or 11 was detected in 96.3% of 108 genital swabs taken from heterosexual men with AGWs attending sexual health clinics in South Africa [48]. Among 74 specimens taken from penile warts of HIV+ men in South Africa, HPV 6 was detected in 42.5%, HPV 11 in 32.9% and HPV 6/11 in 68.5% [53].

### Risk factors for AGWs

Only 2 studies (one among women and another among men) reported on risk factors for AGWs. Among women, the risk of prevalent AGWs was 5 times higher among HIV-1+ than HIV-1- women and 3 times higher among women who smoked cigarettes than those who did not. Among HIV-1+ women with low CD4+ count (≤ 200 cells/μL), the risk of incident AGWs was elevated 20 fold, and 6fold for women with CD4+ count >200 cells/μL. Other risk factors for incident AGWs in women include detection of HPV 6, concurrent bacterial vaginoses, genital ulceration, presence of abnormal cervical cytology and the detection of cervical HPV 52 [44]. Lack of circumcision and HIV infection were risk factors for AGWs in men [45].

## Discussion

To the best of our knowledge this is the first systematic review of the epidemiology of AGWs in SSA. The literature suggests that AGWs are prevalent among both men and women populations seeking care in their respective health care systems. The fewer studies among men is not surprising given that women generally have more frequent contact with the health care system than men. Although there is no marked difference between regions, absence of a standardized protocol for diagnosis might have contributed to the observed variations across studies within the same region. Overall, the prevalence rates were higher than those reported from retrospective administrative databases or medical chart reviews in high income countries possibly because of underlying HIV infection in several studies [54].

Consistent with published studies, the risk for AGWs was higher for HIV+ than HIV- men and women [55]. HIV+ women had almost 2 fold risk for HPV infection than HIV-women. While some AGWs may have been a result of new infections, recrudescence of existing HPV infection has been reported among sexually inactive HIV+ women [56]. Impaired CD4+ T-lymphocyte response and other forms of immune dysfunction may be responsible for altering the natural history of HPV infection among HIV infected individuals [57]. The use of highly active anti-retroviral therapy has been shown to reduce the risk of opportunistic malignancies such as Kaposi sarcoma among HIV+ individuals [58], however, their impact on AGWs remains unclear [55,57,59]*.* On the other hand consistent use of male condoms appears to reduce the risk by 60-70% [60].

Consistent with other studies, HPV 6 and 11 alone or in combination were detected in the few studies that examined HPV genotypes in AGWs specimens albeit small sample sizes. However, the contribution of HPV 11 to the development of AGWs remains unclear [4,7]. The concurrent detection of HPV 52 with HPV 6 was not surprising as co-infection with high risk HPV types has been reported in 20-50% of AGWs [61,62].

In the absence of a clinical test to establish sub clinical HPV 6 and 11 infections, identification of risk factors for acquisition of AGWs independent of other STDs is complex. Consistent with other studies, low CD4+ cell count (≤ 200 cells/μL) and abnormal cervical and anal dysplasias are risk factors for AGWs in HIV+ women and men, respectively [63,64]. Other risk factors for AGWs in women identified in this review included co-infection with HPV 52, and concurrent bacteria vaginoses [65]. In men, anal HPV infection and related dysplasias [39] and lack of circumcision [45] were additional risk factors.

Although AGWs are not life threatening, they cause significant psychological distress and are refractory to conventional therapies, hence the need for prevention [4,66]. The quadrivalent HPV vaccine, correct and consistent condom use and limiting the number of sexual partners are some of the prevention options available to reduce the risk of contracting AGWs.

It is important to note that there are limitations to this study. This review focused only on peer reviewed English language articles published from a few SSA countries, which limits generalization of the findings. Secondly, most studies were conducted in hospital-based study populations, which would favor higher rates than in the general population. Thirdly, the rates should be interpreted with caution because of the differences in study populations and age group studied. While some studies included all adults [31,39], others focused on narrow age ranges of specific populations like young people and pregnant women [23,25] that could have resulted in the observed high rates. Nevertheless, the review provides vital baseline data against which the impact of HPV vaccination could be evaluated in future.

## Conclusions

AGWs are common among selected populations particularly HIV+ men and women. However, there is need for population-based studies on AGWs that will guide policies on effective prevention, treatment and control services.

## Competing interests

The authors declare that they have no competing interests.

## Authors’ contributions

CB conceived the study, searched the literature, drafted the manuscript and produced the final tables FMM, JO, AKB, SK, EKM made substantial contributions to the manuscript and contributed to data interpretation. All authors read and approved the final manuscript.

## References

[B1] Lacey C Therapy for human papillomavirus-related disease J Clin Virol 2005 32 Suppl 1 S82 S90 15753016 10.1016/j.jcv.2004.10.020

[B2] Kjaer SK Tran T Sparen P Tryggvadottir L Munk C Dasbach E Liaw KL Nygård J Nygård M The burden of genital warts: a study of nearly 70,000 women from the general female population in th 4 Nordic countries J Infect Dis 2007 196 1447 1454 10.1086/522863 18008222

[B3] Kliewer EV Demers AA Elliott L Lotocki R Butler JR Brisson M Twenty-year trends in the incidence and prevalence of diagnosed anogenital warts in Canada Sex Transm Dis 2009 36 6 380 386 10.1097/OLQ.0b013e318198de8c 19556932

[B4] Lacey C Lowndes C Shah KV Chapter 4: Burden and management of non-cancerous HPV-related condition: HPV 6/11 Vaccine 2006 24S3 S3:/35 S3/41 16950016 10.1016/j.vaccine.2006.06.015

[B5] Arimi Y Winer RL Feng QA Hughes JP Lee SK Stern ME O'Reilly SF Koutsky LA Development of genital warts after incident detection of human papillomavirus infection in young men J Infect Dis 2010 202 1181 1184 10.1086/656368 20812849

[B6] Anic GM Lee GH Stockwell H Rollison DE Wu Y Papenfuss MR Villa LL Lazcano-Ponce E Gage C Silva RJ Baggio ML Quiterio M Salmeron J Abrahamsen M Giuliano AR Incidence and human papillomavirus (HPV) type distribution of genital warts in a multinational cohort of men: The HPV in men study J Infect Dis 2011 204 1886 1892 10.1093/infdis/jir652 22013227 PMC3209812

[B7] Garland SM Stenben M Sings HL James M Lu S Railkar R Barr E Haupt RM Joura EA Natural history of genital warts: analysis of the placebo arm of 2 randomized phase III trials of a quadrivalent human papillomavirus (types 6, 11, 16, and 18) vaccine J Infect Dis 2009 199 805 814 10.1086/597071 19199546

[B8] Giuliano AR Tortolero-Luna G Ferrer E Burchell AN De Sanjose S Kjaer SK Munoz N Schiffman M Bosch FX Epidemiology of human papillomavirus infection in men, cancers other than cervical and benign conditions Vaccine 2008 26 Suppl 10 K17 K28 18847554 10.1016/j.vaccine.2008.06.021 PMC4366004

[B9] The Lancet Financing HPV vaccination in developing countries Lancet 2011 377 9777 1544 21550467 10.1016/S0140-6736(11)60622-3

[B10] Donovan B Franklin N Guy R Grulich AE Regan DG Ali H Wand H Fairley CK Quadrivalent human papillomavirus vaccination and trends in genital warts in Australia: analysis of national sentinel surveillance data Lancet Infect Dis 2011 11 39 44 10.1016/S1473-3099(10)70225-5 21067976

[B11] Kreiss J Kiviat N Plummer F Roberts P Waiyaki P Ngugi E Holmes KK Human Immunodeficiency Virus, Human papillomavirus, and cervical intraepithelial neoplasis in Nairobi prostitutes Sex Transm Infect 1992 19 1 54 59 10.1097/00007435-199201000-00011 1313992

[B12] Fonck K Kidula N Kirui P Ndinya-Achola J Bwayo J Claeys P Temmerman M Pattern of sexually transmitted diseases and risk factors among women attending an STD referral clinic in Nairobi, Kenya Sex Transm Dis 2000 7 417 423 10949433 10.1097/00007435-200008000-00007

[B13] Mayaud P Weiss H Lacey C Uledi E Kopwe LT Ka-Gina G Grosskurth H Hayes RJ Mabey DC Lacey CJ The interrrelation of HIV, cervical human papillomavirus, ans neoplasia among antenatal clinic attenders in Tanzania Sex Transm Infect 2001 77 4 248 254 10.1136/sti.77.4.248 11463923 PMC1744347

[B14] Riedner G Hoffmann O Nichombe F Lyamuya EM Mmbando D Maboko L Hay P Todd J Hayes R Hoelscher M Grosskurth H Baseline survey of sexually transmitted infections in a cohort of female bar workers in Mbeya region, Tanzania Sex Transm Infect 2003 79 382 387 10.1136/sti.79.5.382 14573833 PMC1744739

[B15] Namkinga L Matee M Kivaisi A Moshiro C Prevalence and risk factors for vaginal candidiasis among women seeking primary care for genital infections in Dar es Salaam, Tanzania East Afr Med J 2005 82 3 138 143 16122076 10.4314/eamj.v82i3.9270

[B16] Amone-P'Olak Psychological impact of wae and sexual abuse on adolescent girls in northern Uganda Intervention 2005 3 1 33 45

[B17] Mbizvo E Msuya S Hussain A Chirenje M Mbizvo M Sam N Stray-Pedersen B HIV and sexually transmitted infections among women presenting at urban primary health care clinics in two cities of sub-Saharan Africa Afr J Reprod Health 2005 9 1 88 98 10.2307/3583163 16104658

[B18] Msuya S Mbizvo E Stray-Pedersen B Sundby J Sam N Hussain A Risk assessment at the primary health care level in Moshi, Tanzania: limits in predicting sexually transmitted infections among women Cent Afr J Med 2006 52 9–12 97 104 20353133 10.4314/cajm.v52i9-12.62593

[B19] Riedner G Rusizoka M Mmbando D Maboko L Grosskurth H Todd J Hayes R Hoelscher M Decline in sexually transmitted infection prevalence and HIV incidence in female barworkers attending prevention and care services in Mbeya Region Tanzania. AIDS 2006 20 4 609 615 10.1097/01.aids.0000210616.90954.47 16470126

[B20] Aboud S Msamanga G Read JS Mwatha A Chen YQ Potter D Potter D Valentine M Sharma U Hoffmann I Taha TE Goldenberg RL Fawzi WW Genital tract infections amon HIV-infected pregnant women in Malawi, Tanzania and Zambia Int J STD AIDS 2008 19 12 824 832 10.1258/ijsa.2008.008067 19050213 PMC2698963

[B21] Banura C Franceschi S van Doorn L Arslan A Kleter B Wabwire-Mangen F Mbidde EK Quint W Weiderpass E Infection with human papillomavirus and HIV among young women in Kampala, Uganda J Infect Dis 2008 197 4 555 562 10.1086/526792 18237268

[B22] Banura C Franceschi S van Doorn L Arslan A Kleter B Wabwire-Mangen F Mbidde EK Quint W Weiderpass E Prevalence, incidence and clearance of human papillomavirus infection among young primiparous pregnant women in Kampala, Uganda Int J Cancer 2008 123 9 2180 2187 10.1002/ijc.23762 18711697

[B23] Urassa W Moshiro C Chalamilla G Mhalu F Sandstrom E Risky sexual practices among youth attending a sexually transmitted infection clinic in Dar es Salaam, Tanzania BMC Infect Dis 2008 8 159 10.1186/1471-2334-8-159 19019224 PMC2596153

[B24] Grijsen ML Graham S Mwangome M Githua P Mutimba S Wamuyu L Okuku H Price MA McClelland RS Smith AD Sanders EJ Screening for genital and anorectal sexually transmitted infections in HIV prevention trials in Africa Sex Transm Infect 2008 84 364 370 10.1136/sti.2007.028852 18375645 PMC3895478

[B25] Mapingure MP Msuya S Kurewa NE Mujoma MW Sam N Chirenje MZ Rusakaniko S Saugstad LF de Vlas SJ Stray-Pedersen B Sexual behavior does not reflect HIV-1 prevalence differences: a comparison study of Zimbabwe and Tanzania J International AIDS Society 2010 13 45 10.1186/1758-2652-13-45 PMC2997084 21080919

[B26] Latif A Bvumbe J Muongerwa J Paraiwa E Chikosi W Sexually transmitted diseases in pregnant women in Harare, Zimbabwe Afr j Sex Transm Dis 1984 1 1 21 23 12340184

[B27] Mason P Gwanzura L Latif A Marowa E Genital infections in women attending a genito-urinary clinic in Harare, Zimbabwe Genitourin Med 1990 66 178 181 2196216 10.1136/sti.66.3.178 PMC1194498

[B28] Kristensen J The prevalence of symptomatic sexually transmitted diseases and human immunodeficiency virus infection in outpatients in Lilongwe, Malawi Genitourin Med 1990 66 4 244 246 2391110 10.1136/sti.66.4.244 PMC1194521

[B29] Nzila N Laga M Thiam MA Mayimona K Edidi B Van Dyck E Behets F Hassig S Nelson A Mokwa K HIV and other sexually transmitted diseases among female prostitutes in Kinshasa AIDS 1991 5 715 721 10.1097/00002030-199106000-00011 1883543

[B30] Le Bacq F Mason P Gwanzura L Robertson V Latif A HIV and other sexually transmitted diseases at a rural hospital in Zimbabwe Genitourin Med 1993 69 352 356 8244351 10.1136/sti.69.5.352 PMC1195116

[B31] Maher D Hoffman I Prevalence of genital infections in medical inpatients in Blantyre, Malawi J Infect 1995 31 77 78 8522842 10.1016/s0163-4453(95)91674-1

[B32] Taha T Dallabetta GA Hoover DR Chiphangwi JD Mtimavalye LA Liomba GN Kumwenda NI Miotti PG Trends of HIV-1 and sexually transmitted diseases among pregnant and post-partum women in urban Malawi AIDS 1998 12 197 203 10.1097/00002030-199802000-00010 9468369

[B33] Klaskala W Brayfield BP Kankasa C Bhat G West JT Mitchell CD Wood C Epidemiological Characteristics of Human Herpesvirus-8 Infection in a Large Population of Antenatal Women in Zambia J Med Virol 2005 75 93 100 10.1002/jmv.20242 15543582

[B34] Kurewa N Mapingure M Munjoma M Chirenje M Rusakaniko S Stray-Pedersen B The burden and risk factors for sexually transmitted infections and reproductive tract infections among pregnant women in Zimbabwe BMC Infect Dis 2010 10 127 10.1186/1471-2334-10-127 20492681 PMC2881092

[B35] Menendez C Castellsague X Renom M Sacarlal J Quinto L Lloveras B Klaustermeier J Kornegay JR Sigauque B Bosch FX Alonso PL Prevalence and risk factors of sexually transmitted infections and cervical neoplasia in women from a rural area of Southern Mozambique Infect Dis Obstet Gynecol 2010 Published online 2010 July 11 10.1155/2010/609315 PMC2913799 20706691

[B36] Oni A Adu F Ekweozor C Isolation of herpes simplex virus from sexually transmitted disease patients in Ibadan, Nigeria Sex Transm Dis 1994 21 4 187 190 10.1097/00007435-199407000-00001 7974067

[B37] Ghys PD Diallo OM Ettiègne-Traoré V Yeboue KM Gnaore E Lorougnon F Kal K Van Dyck E Brattegaard K Hoyi YM Whitaker JP De Cock KM Greenberg AE Piot P Laga M Genital ulcers associated with human immunodeficiency virus-related immunosuppression in female sex workers in Abidjan, Ivory Coast J Infect Dis 1995 172 5 1371 1374 10.1093/infdis/172.5.1371 7594681

[B38] Meda N Sangaré L Lankoandé S Sanou P Campaore PT Catraye J Cartoux M Soudré RB Pattern of sexually transmitted diseases among pregnant women in Burkina Faso, West Africa: potential for a clinical management based on simple approaches Genitourin Med 1997 73 188 193 9306899 10.1136/sti.73.3.188 PMC1195819

[B39] Okesola A Fawole O Prevalence of human papillomavirus genital infections in sexually transmitted diseases clinic attendees in Ibadan West Afr J Med 2000 19 3 195 199 11126083

[B40] Bakare RA Oni AA Umar US Adewole IF Shokunbi WA Fayemiwo SA Fasina NA Pattern of sexually transmitted diseases among commercial sex workers (CSWs) in Ibadan, Nigeria Afr J Med Med Sci 2002 31 3 243 247 12751565

[B41] Domfeh AB Wiredu EK Adjei AA Ayeh-Kumi PFK Adiku TK Tet-tey Y Gyasi RK Armah HB Cervical human papillomavirus infection in Accra, Ghana Ghana Med J 2008 42 2 71 78 19180207 10.4314/gmj.v42i2.43596 PMC2631263

[B42] Sagay AS Imade GE Onwuliri V Egah DZ Grigg MJ Musa J Thacher TD Adisa JO Potts M Short RV Genital tract abnormalities among female sex workers who douche with lemon/lime juice in Nigeria Afr J Reprod Health 2009 13 1 37 45 20687264

[B43] Jombo E Okwori E Otor G Odengle E Patterns of genital ulcer diseases among HIV/AIDS patients in Benue State, North Central Nigeria Internet J Epidemiol 2009 7 2

[B44] Low A Clayton T Konate I Nagot N Ouedraogo A Huet C Didelot-Rousseau M-N Michel S Van de Perre P Mayaud P the Yérélon Cohort Study Group, Genital warts and infection with human immunodeficiency virus in high-risk women in Burkina Faso: a longitudinal study BMC Infect Dis 2011 11 20 10.1186/1471-2334-11-20 21251265 PMC3031229

[B45] Smith J Moses S Hudgens MG Parker CB Agot K Maclean I Ndinya-Acholi JO Snijders P Meijer JF C. J. L. M Bailey RC Increased risk of HIV acquisition among Kenyan men with human papillomavirus infection JID 2010 201 11 1677 1685 10.1086/652408 20415595 PMC2873838

[B46] Tobian AA Grabowski MK Kigozi G Gravitt PE Eaton KP Serwadda D Nalugoda F Wawer MJ Quinn TC Gray RH High-risk human papillomavirus prevalence is associated with HIV infection among heterosexual men in Rakai, Uganda Sex Transm Infect 2012 89 2 122 127 22628661 10.1136/sextrans-2012-050524 PMC3640492

[B47] Machekano R Bassett M Zhou P Mbizvo M Latif A Katzenstein D Report of sexually transmitted diseases by HIV infected man during follow up: time to target the HIV infected? Sex Transm Inf 2000 76 186 192 10.1136/sti.76.3.188 PMC1744145 10961196

[B48] Müller EE Chirwa TF Lewis DA Human papillomavirus infection in heterosexual South African men attending sexual health services: associations between HPV and HIV serostatus Sex Transm Infect 2010 86 175 180 10.1136/sti.2009.037598 19880970

[B49] Wade AS Kane CT Diallo PA Diop AK Gueye K Mboup S Ndoye I Lagarde E HIV infection and sexually transmitted infections among men who have sex with men in Senegal AIDS 2005 19 2133 2140 10.1097/01.aids.0000194128.97640.07 16284463

[B50] Lavreys L Rakwar J Thompson M Jackson D Mandaliya K Chohan BH Bwayo JJ Ndinya-Ochola JO Kreiss JK Effect of circumcision on incidence of HIV-1 and other sexually transmitted diseases: A prospective cohort study of trucking company employees in Kenya J Infect Dis 1999 180 2 330 336 10.1086/314884 10395846

[B51] Ozumba B Megafu U Pattern of vulval warts at the University of Nigeria teaching hospital, Enugu, Nigeria Int J Gynecol Obstet 1991 34 347 352 10.1016/0020-7292(91)90603-3 1674482

[B52] Wright C Taylor L Cooper K HPV typing of vulvovaginal condylomata in children S Afr Med J 1995 85 10 Suppl 1096 1101 8914560

[B53] Firnhaber C Sello M Maskew M Williams S Schulze D Williamson AL Allan B Lewis D Human papillomavirus types in HIV seropositive men with penile warts in Johannsesburg, South Africa Int J STD AIDS 2011 22 2 107 109 10.1258/ijsa.2010.010306 21427434

[B54] Patel H Wagner M Singhal P Kothari S Systematic review of the incidence and prevalence of genital warts BMC Infect Dis 2013 13 39 10.1186/1471-2334-13-39 23347441 PMC3618302

[B55] Silverberg M Ahdieh LM Anastos K Burk R Cu-Uvin SD Greenblatt RM Klein RS Massad S Minkoff H Murderspach L Palefsky J Piessens E Schuman P Watts H Shah KV The impact of HIV infection and immunodeficiency on human papillomavirus type 6 or 11 infection and on genital warts Sex Transm Dis 2002 29 427 435 10.1097/00007435-200208000-00001 12172526

[B56] Strickler H Burk RD Fazzari M Burk R Fazzari M Anostos K Minkkoff H Massad LS Hall C Bacon M Levine AM Watts DH Silverberg MJ Xue X Schlecht NF Melnick S Palefsky JM Natural history and possible reactivation of human Strickler H Natural history and possible reactivation of human papillomavirus in human immunodeficiency virus-positive women J Natl Cancer Inst 2005 97 577 586 10.1093/jnci/dji073 15840880

[B57] Massad L Silverberg M Springer G Minkoff H Hessol N Palefsky JM Strickler HD Levine AM Sacks HS Moxley M Heather WD Effect of antiretroviral therapy on incidence of genital warts and vulvular neoplasia among women with human immunodeficiency virus Am J Obstet Gynecol 2004 190 1241 1248 10.1016/j.ajog.2003.12.037 15167825

[B58] Nasti G Martellota F Berretta M Mena M Fasan M Di Perri G Impact of highly active anti retroviral therapy on the presenting features and outcome of patients with acquired immunodeficiency syndromme-related Kaposi sarcoma Cancer 2003 11 2440 2446 14635079 10.1002/cncr.11816

[B59] Heard I Tassie J Schmitz V Mandelbrot L Kazatchkine M Orth G Increased risk of cervical disease among human immunodeficiency virus-infected women with severe immunosuppression and high human papillomavirus load(1) Obstet Gynecol 2000 96 403 409 10.1016/S0029-7844(00)00948-0 10960634

[B60] Manhart L Koutsky L Do condoms prevent genital HPV infection, external genital warts or cervical neoplasia? Sex Transm Dis 2002 29 11 725 735 10.1097/00007435-200211000-00018 12438912

[B61] Aubin F Prétet JL Jacquard AC Saunier M Carcopino X Jaroud F Pradat P Soubeyrand B Leocmach Y Mougin C Riethmuller D EDiTH Study Group Human papillomavirus genotype distribution in external acuminata condylomata: a Large French National Study (EDITH IV) Clin Infect Dis 2008 47 610 615 10.1086/590560 18637758

[B62] Vandepapeliere P Barraso R Meijer CJ Walboomers JM Wettendorff M Stanberry LR Lacey CJ Randomized controlled trial of an adjuvented human papillomavirus type 6 L2E7 vaccine: infection of external genital warts with multiple HPV types and failure of therapeutic vaccination J Infect Dis 2005 192 2099 2107 10.1086/498164 16288373

[B63] Wen L Estcourt C Simpson J Mindel A Risk factors for acqusition of genital warts: are condoms protective? Sex Transm Infect 1999 75 312 316 10.1136/sti.75.5.312 10616354 PMC1758246

[B64] Moscicki A Hills N Shiboski S Powell K Jay N Hanson E Miller S Clayton L Farhat S Broering J Darragh T Palefsky J Risks for incident human papillomavirus infection and low-grade squamous intraepithelial lesion development in young females JAMA 2001 285 2995 3002 10.1001/jama.285.23.2995 11410098

[B65] Watts DH Fazzari M Minkoff H Hillier SL Sha B Glesby M Levine AM Burk R Palefsky JM Moxley M Ahdieh-Grant L Strickler HD Effects of bacterial vaginosis and other genital infections on the natural history of human papillomavirus infection in HIV-1 infected and high-risk HIV-1 uninfected women J Infect Dis 2005 191 7 1129 1139 10.1086/427777 15747249

[B66] Persson G Dahlöf LG Krantz I Physical and psychological effects of anogenital warts in female patients Sex Transm Dis 1993 20 10 13 10.1097/00007435-199301000-00003 8430353

